# Supplementary material for: Validation of an admission coagulation panel for risk stratification of COVID-19 patients
Source: PLoS One. 2021 Mar 19;16(3):e0248230. doi: 10.1371/journal.pone.0248230 (PMC7979266; doi:10.1371/journal.pone.0248230)
Supplement: S1 Dataset — (PDF) [file pone.0248230.s001.pdf]

Length of S	DD1	PF1	TAT1	FM1	AbnlDDime	DD>2000	DD>3000	#Abnl MOC
34.00	1630.00	219	9	9	1	0	0	3.00
11.00	5652.00	178	7.4	15	1	1	1	3.00
26.00	974.00	647	13.3	18	1	0	0	4.00
7.00	2490.00	449	18.5	78	1	1	0	4
13.00	60000.00	1118	60	130	1	1	1	4
15.00	30035.00	1200	60	150	1	1	1	4
21.00	60000.00	753	44.9	32	1	1	1	4
4.00	15568.00	283	12	32	1	1	1	3
10.00	3257.00	277	14	55	1	1	1	3
10.00	1823.00	470	9.8	7	1	0	0	4
21.00	60000.00	1200	46	150	1	1	1	4
19.00	972.50	1200	60	21	1	0	0	4.00
46.00	2055.00	585	14.9	18	1	1	0	4
11.00	60000.00	1014	39.7	110	1	1	1	4
16.00	1566.00	151	11.1	15	1	0	0	3
6.00	930.00	339	5.6	7	1	0	0	3
13.00	1647.00	580	6.1	11	1	0	0	4
15.00	609.00	228	7.6	6.99	1	0	0	2.00
52.00	1728.50	168	13.8	7	1	0	0	3
30.00	1492.00	151	10.3	10	1	0	0	3.00
43.00	772.50	103	5.1	8	1	0	0	2.00
8.00	739.00	464	29.9	7	1	0	0	3
13.00	1950.00	529	18.5	7	1	0	0	4
13.00	734.50	289	5	6.99	1	0	0	2.00
23.00	1593.00	374	14.2	8	1	0	0	4.00
14.00	784.00	266	6.2	7	1	0	0	2
16.00	974.00	286	9.3	7	1	0	0	3.00
36.00	850.00	430	4.9	10	1	0	0	3.00
ongoing	1350.00	109	6.6	12	1	0	0	3.00
17.00	223.00	97	6.7	7	0	0	0	1
15.00	277.00	291	20.6	7	0	0	0	3
16.00	917.00	225	5.4	6.99	1	0	0	1.00
29.00	352.00	163	3.7	10	0	0	0	1
17.00	1311.00	708	26.8	150	1	0	0	4.00
25.00	3835.00	557	28.4	53	1	1	1	4
35.00	40684.00	486	41.5	150	1	1	1	4
45.00	1698.00	386	18.7	22	1	0	0	4.00
27.00	5295.00	764	9.6	14	1	1	1	4.00
21.00	899.00	513	38.4	35	1	0	0	4
69.00	609.00	117	3.1	7	1	0	0	1
30.00	4948.00	487	13.2	10	1	1	1	4
28.00	1453.00	119	3	15	1	0	0	2.00
36.00	14431.00	972	31.9	25	1	1	1	4
50.00	372.00	92	4	7	0	0	0	0
ongoing	702.00	287	14.8	150	1	0	0	3.00
50.00	1691.00	498	7.9	6.99	1	0	0	3.00

42.00	718.00	292	5.6	6.99	1	0	0	3.00
27.00	1024.00	208	6.5	6.99	1	0	0	2.00
35.00	556.00	344	2.6	6.99	0	0	0	1.00
43.00	3544.00	232	35.2	14	1	1	1	3.00
42.00	2054.00	250	14.6	22	1	1	0	3.00
22.00	1634.00	151	4	86	1	0	0	2.00
12.00	772.00	466	8.5	11	1	0	0	4.00
11.00	992.00	508	20.7	19	1	0	0	4.00
26.00	7535.00	646	60	150	1	1	1	4.00
11.00	493.50	144	5.1	7	0	0	0	1.00
15.00	433.50	127	3.8	12	0	0	0	1.00
15.00	60000.00	737	14.9	95	1	1	1	4.00
6.00	991.00	191	11.6	10	1	0	0	3.00
9.00	478.00	206	7.8	6.99	0	0	0	1.00
7.00	313.00	152	2.6	7	0	0	0	1.00
7.00	383.00	165	10.8	6.99	0	0	0	1.00
4.00	555.00	188	5.6	6.99	0	0	0	1.00
5.00	507.50	179	6.5	8	0	0	0	2.00
6.00	989.00	379	6.5	6.99	1	0	0	3.00
7.00	1068.00	844	60	8	1	0	0	4.00
9.00	344.00	185	7	6.99	0	0	0	1.00
11.00	535.00	132	3.8	22	0	0	0	1.00
5.00	465.00	259	9.9	7	0	0	0	2.00
7.00	1291.50	279	10.2	10	1	0	0	3.00
8.00	4720.00	1065	60	119	1	1	1	4.00
4.00	1087.00	305	4.2	6.99	1	0	0	2.00
7.00	750.00	174	4	29	1	0	0	2.00
8.00	1693.00	261	10.2	14	1	0	0	3.00
8.00	648.00	373	12.4	6.99	1	0	0	3.00
2.00	329.00	238	9.7	6.99	0	0	0	1.00
4.00	466.50	478	3	6.99	0	0	0	1.00
4.00	557.50	89	6.1	6.99	0	0	0	1.00
4.00	629.00	198	4.5	8	1	0	0	2.00
4.00	28774.00	309	3.4	131	1	1	1	3.00
5.00	302.00	226	6.2	6.99	0	0	0	1.00
5.00	1207.00	266	5.7	7	1	0	0	3.00
5.00	596.00	156	3.3	8	1	0	0	2.00
5.00	617.00	240	4.9	6.99	1	0	0	1.00
5.00	600.00	395	5.6	11	1	0	0	4.00
6.00	3193.00	834	18	9	1	1	1	4.00
6.00	795.00	164	5.4	6.99	1	0	0	1.00
6.00	395.00	170	4.7	9	0	0	0	1.00
6.00	963.50	366	7.6	6.99	1	0	0	3.00
6.00	950.00	398	2.7	6.99	1	0	0	2.00
6.00	3250.00	602	7.6	6.99	1	1	1	3.00
6.00	905.00	269	2.9	6.99	1	0	0	1.00
6.00	825.00	77	4.1	8	1	0	0	2.00

6.00	234.00	166	3.9	8	0	0	0	1.00
7.00	1045.50	135	4.8	6.99	1	0	0	1.00
7.00	888.50	359	8.7	6.99	1	0	0	3.00
7.00	734.00	171	4	8	1	0	0	2.00
7.00	324.00	105	4.3	7	0	0	0	1.00
7.00	410.00	251	3.7	8	0	0	0	1.00
8.00	665.00	259	9.2	6.99	1	0	0	2.00
8.00	1020.00	125	3.1	6.99	1	0	0	1.00
8.00	732.00	354	5.9	6.99	1	0	0	3.00
9.00	1119.00	742	60	27	1	0	0	4.00
9.00	503.00	311	9.6	7	0	0	0	3.00
10.00	377.00	219	5.2	8	0	0	0	1.00
10.00	1219.00	898	60	60	1	0	0	4.00
11.00	532.50	175	7.9	6.99	0	0	0	1.00
12.00	766.00	22	7.3	10	1	0	0	3.00
14.00	220.00	233	6.1	6.99	0	0	0	1.00
15.00	1039.00	163	8.1	6.99	1	0	0	2.00
16.00	1871.00	331	6.6	7	1	0	0	4
ongoing	37233.00	998	60	150	1	1	1	4.00
ongoing	2423.00	170	9.9	10	1	1	0	3.00
10.00	643.00	231	3.5	6.99	1	0	0	1.00
13.00	895.00	276	7.1	6.99	1	0	0	2.00
13.00	741.00	191	7.7	6.99	1	0	0	2.00
16.00	1100.00	227	6.2	6.99	1	0	0	2.00
22.00	7028.00	20	20.5	60	1	1	1	3.00
17.00	1935.00	378	43.2	8	1	0	0	4.00
25.00	600.00	163	5	10	1	0	0	2.00
15.00	1090.00	173	15.7	9	1	0	0	3.00
14.00	889.00	192	4.8	9	1	0	0	2.00
34.00	7613.00	972	37.5	35	1	1	1	4.00
23.00	1283.00	329	5.4	7	1	0	0	3.00
19.00	3209.00	570	14.1	15	1	1	1	4.00
37.00	744.00	277	3.9	6.99	1	0	0	1.00
55.00	513.00	223	8	9	0	0	0	2.00
11.00	914.00	154	6.5	6.99	1	0	0	2.00
7.00	4883.00	334	11.6	23	1	1	1	4.00
4.00	616.00	147	2.8	8	1	0	0	2.00
5.00	1121.00	351	6.6	13	1	0	0	4.00
6.00	12842.00	654	13.2	63	1	1	1	4.00
12.00	414.00	264	8.5	6.99	0	0	0	1.00
8.00	729.00	416	10.4	7	1	0	0	4.00
23.00	864.00	192	5.6	6.99	1	0	0	2.00
81.00	1178.00	199	7.9	7	1	0	0	2
34.00	891.00	308	16.9	7	1	0	0	4
39.00	220.00	82	4.6	7	0	0	0	0
10.00	1301.00	161	5.6	7	1	0	0	2
1.00	288.00	205	7.1	7	0	0	0	3

2.00	1005.00	140	2	7	1	0	0	1
2.00	588.00	131	2	7	1	0	0	1
2.00	620.00	140	2.9	8	1	0	0	2
2.00	392.00	235	9.4	7	0	0	0	1
3.00	220.00	253	8	7	0	0	0	1
3.00	615.00	489	9.8	31	1	1	1	1
3.00	12575.00	274	8.2	7	1	0	0	4
3.00	772.00	410	29.7	35	1	0	0	3
3.00	768.00	199	3.2	8	1	0	0	2
3.00	656.00	159	3.7	7	0	0	0	1
3.00	4318.00	412	9	10	1	1	1	4
3.00	674.00	122	2.2	7	1	0	0	1
3.00	775.00	152	5.1	7	1	0	0	1
3.00	475.00	309	7.1	7	1	0	0	2
3.00	1596.00	179	2.2	8	1	0	0	2
3.00	737.00	125	6.9	7	1	0	0	2
3.00	548.00	292	3.2	7	0	0	0	2
3.00	516.00	191	4	25	0	0	0	1
4.00	337.00	756	60	150	0	0	0	4
4.00	503.00	341	3.8	7	0	0	0	1
4.00	577.00	126	4.3	7	1	0	0	1
4.00	815.00	345	6.4	7	1	0	0	3
4.00	877.00	175	2	10	1	0	0	2
4.00	852.00	299	15	150	1	0	0	4
4.00	286.00	104	4.5	14	0	0	0	1
4.00	1209.00	213	5.6	7	1	0	0	2
4.00	253.00	186	7.5	7	0	0	0	1
5.00	34430.00	748	18.9	150	1	1	1	4
5.00	7198.00	335	5.9	28	1	1	1	4
5.00	510.50	330	6.1	9	0	0	0	3
5.00	897.00	654	54.5	28	1	0	0	4
6.00	645.00	206	2	7	0	0	0	1
6.00	1966.00	251	12.1	9	1	0	0	3
6.00	2179.00	308	3.1	7	1	1	0	2
6.00	4075.00	882	18.4	92	1	1	1	4
6.00	1189.00	117	7.3	7	1	0	0	2
6.00	985.00	274	6	9	1	0	0	3
6.00	4851.00	1200	56.7	17	1	1	1	4
6.00	965.00	826	4.2	7	1	0	0	2
6.00	1620.00	369	5	9	1	0	0	3
6.00	892.00	165	4.2	7	1	0	0	1
6.00	659.50	140	8.3	74	0	0	0	3
7.00	4627.00	839	60	98	1	1	1	4
7.00	2233.00	302	36.5	12	1	1	1	4
7.00	855.00	146	5.6	7	1	0	0	3
7.00	713.00	209	2	7	0	0	0	1
7.00	819.00	170	2	17	1	0	0	2

7.00	1668.00	231	2	7	1	0	0	1
7.00	1585.00	397	3.4	58	1	0	0	3
7.00	612.00	111	6.4	7	1	0	0	2
7.00	945.00	194	5.3	11	1	0	0	2
7.00	336.00	197	10.8	7	0	0	0	1
8.00	503.00	134	3.4	8	0	0	0	1
8.00	1935.00	670	12.6	9	1	1	0	4
8.00	1390.00	340	37.9	71	1	0	0	4
8.00	2240.00	214	11	64	1	1	0	3
8.00	449.00	371	27.9	7	0	0	0	2
8.00	7606.00	651	40.8	21	1	1	1	4
8.00	678.00	284	2	7	1	0	0	1
8.00	311.00	112	9.4	7	0	0	0	1
8.00	1140.00	270	6.2	31	1	0	0	3
8.00	823.00	647	59.3	122	0	0	0	4
8.00	695.00	288	11.5	17	1	0	0	3
8.00	763.50	299	7.2	7	1	0	0	4
8.00	246.00	161	13.8	7	0	0	0	1
8.00	830.00	162	7.4	7	1	0	0	2
8.00	726.50	158	2.8	7	1	0	0	1
9.00	1902.00	793	10.4	8	1	0	0	4
9.00	440.00	228	5.4	7	0	0	0	0
9.00	338.00	283	7.4	7	0	0	0	1
9.00	503.00	436	10.5	9	1	0	0	3
9.00	897.00	201	3.4	7	1	0	0	1
9.00	235.00	148	5.3	7	0	0	0	1
9.00	925.50	243	4.6	9	1	0	0	2
9.00	356.00	124	3.1	9	0	0	0	1
9.00	2025.00	451	16.5	9	1	1	0	4
9.00	741.00	132	3.7	7	1	0	0	1
9.00	895.00	247	5	7	1	0	0	1
9.00	672.00	322	27.2	12	1	0	0	4
10.00	590.00	150	4.6	7	1	0	0	2
11.00	465.00	206	4	7	0	0	0	0
11.00	582.00	224	6.6	7	0	0	0	2
11.00	1889.00	735	60	16	1	0	0	4
11.00	1776.00	262	3.6	11	1	0	0	2
11.00	1311.00	392	7.4	7	1	0	0	3
13.00	2224.00	20	10.6	8	1	1	0	3
13.00	2245.00	552	12.7	18	1	1	0	4
13.00	787.00	101	2	7	0	0	0	2
13.00	20555.00	320	13.3	150	1	1	1	4
13.00	635.00	395	4.5	7	1	0	0	2
13.00	5196.00	35	14.4	89	1	1	1	3
13.00	780.00	220	9.3	7	1	0	0	2
13.00	654.00	282	7.8	7	1	0	0	3
13.00	479.00	244	4.7	7	0	0	0	0

14.00	1078.00	315	5.5	8	1	0	0	3
15.00	583.00	164	6.1	102	0	0	0	3
15.00	859.00	161	10.7	7	1	0	0	2
15.00	18662.00	264	7.4	20	1	1	1	3
15.00	688.00	115	7.4	7	1	0	0	3
15.00	1257.00	318	5.6	7	1	0	0	3
15.00	473.00	120	7.6	7	0	0	0	1
16.00	3060.00	153	14	15	1	1	1	3
16.00	929.00	253	7.1	7	1	0	0	2
17.00	708.00	159	3.9	7	0	0	0	2
18.00	2067.00	549	60	23	1	1	0	4
18.00	690.00	121	2.8	7	1	0	0	1
18.00	1049.00	310	16.1	7	1	0	0	3
18.00	1294.00	326	11.8	10	1	0	0	4
19.00	762.00	329	21.1	7	1	0	0	3
20.00	901.00	801	60	148	1	0	0	4
21.00	733.00	527	5.8	15	0	0	0	2
21.00	499.00	73	2	7	0	0	0	0
21.00	1645.00	545	7.1	7	1	0	0	3
21.00	1140.00	575	7.9	8	1	0	0	4
22.00	1345.00	210	6.1	7	1	0	0	2
22.00	377.00	191	6.5	7	0	0	0	1
23.00	18811.00	1200	60	150	1	1	1	4
23.00	559.00	159	2.7	7	0	0	0	0
24.00	720.00	170	4.1	7	0	0	0	1
24.00	870.00	280	3.6	7	1	0	0	1
24.00	1827.00	543	16.2	10	1	0	0	4
24.00	1060.00	284	3.3	8	1	0	0	2
26.00	967.00	707	60	142	1	0	0	4
27.00	3592.00	214	15.2	76	1	0	0	4
27.00	853.00	196	6	12	1	0	0	3
27.00	623.00	278	6.8	7	0	0	0	2
27.00	1565.00	1200	60	19	1	0	0	4
29.00	869.50	232	8.5	13	1	0	0	3
30.00	1551.00	330	2	14	1	0	0	3
33.00	418.00	336	7.1		0	0	0	2
35.00	580.00	180	4.3	12	0	0	0	2
38.00	1328.00	180	24.3	8	1	0	0	3
44.00	519.00	874	60	8	0	0	0	3
45.00	1326.00	144	2	7	1	0	0	2
48.00	770.00	319	7.6	7	1	0	0	3
49.00	634.00	234	7.1	20	1	0	0	3

MoCHA1 H	#AbnlMOC	DaysUntilV	Vasc Endpc	Type	Intubation	Death	Major blee	Age
1.00	2.00	0.00	1	PE, MI	1	0	0	74
1.00	3.00	0.00	1	PE/DVT				53
1.00	3.00	0.00	1	MI	1	0	0	76
1	4	0.00	1	PE	1	0	0	59
2	4	0.00	1	stroke and	1	0	0	69
1	4	0.00	1	DVT	1	0	0	61
1	4	0.00	1	DVT	1	0	0	76
2	3	1.00	1	DVT	0	0	0	62
1	3	1.00	1	DVT	0	0	0	76
1	3	2.00	1	DVT	0	0	0	81
1	4	2.00	1	DVT	1	0	0	71
1.00	3.00	3.00	1	DVT	1	0	0	72
1	4	3.00	1	DVT	1	0	0	66
2	4	3.00	1	DVT	1	0	0	65
1	2	4.00	1	PE	1	1	0	90
1	2	5.00	1	PE	1	1	0	45
1	3	5.00	1	MI	0	0	0	89
1.00	1.00	6.00	1	DVT	0	0		51
1	2	6.00	1	Line Clot	1	0	0	50
2.00	2.00	7.00	1	Line Clot	1	0	1	24
2.00	1.00	7.00	1	Line Clot	1	0	0	66
1	2	7.00	1	DVT	1	1	0	50
1	3	7.00	1	DVT	1	0	0	52
2.00	1.00	8.00	1	PE	0	0	0	76
1.00	3.00	8.00	1	DVT	1	0	0	58
2	1	8.00	1	Line clot	1	1	0	56
2.00	2.00	9.00	1	MI	1	0	1	66
1.00	2.00	10.00	1	Stroke	1	0	0	48
1.00	2.00	10.00	1	DVT	1	0	1	54
1	1	10.00	1	CRRT	1	1	0	52
1	3	11.00	1	DVT	0	0	0	47
1.00	0.00	13.00	1	PE	0	0	0	44
1	1	14.00	1	DVT	1	0	0	29
1.00	3.00	15.00	1	PE	0	0	0	70
1	4	15.00	1	stroke	1	1	1	65
1	4	16.00	1	CRRT	1	0	0	29
1.00	3.00	17.00	1	Stroke	1	0	0	53
1.00	4.00	17.00	1	DVT	0	0	1	42
1	3	19.00	1	Line clot	1	1	1	35
2	0	20.00	1	DVT	1	0	0	28
2	4	23.00	1	DVT	1	1	1	70
1.00	1.00	26.00	1	DVT	1	1	1	70
2	4	26.00	1	stroke	1	0	0	61
1	0	30.00	1	DVT	0	0	0	54
2.00	2.00	52.00	1	DVT	1	0	0	50
3.00	2.00		0		1	0	0	64

2.00	2.00	0	1	0	0	69
1.00	1.00	0	1	0	0	52
1.00	1.00	0	1	0	0	58
1.00	3.00	0	1	0	0	34
1.00	3.00	0	1	0	1	42
1.00	1.00	0	1	1	0	36
1.00	3.00	0	DNR	1	0	82
2.00	3.00	0	0	0	0	76
1.00	4.00	0	0	0	1	77
2.00	1.00	0	0	0	0	57
2.00	1.00	0	0	0		54
1.00	4.00	0	0	0	0	45
1.00	2.00	0	0	0	0	56
1.00	1.00	0	0	0	0	57
1.00	1.00	0	0	0	0	67
1.00	1.00	0	0	0	0	41
1.00	1.00	0	0	0	0	22
1.00	2.00	0	0	0	0	43
1.00	2.00	0	0	0	0	29
2.00	3.00	0	0	0	0	46
2.00	1.00	0	0	0	0	75
3.00	1.00	0	0	0	0	57
1.00	2.00	0	0	0	0	62
1.00	2.00	0	0	0		68
1.00	4.00	0	0	0	0	66
2.00	1.00	0	DNR	1	0	80
2.00	1.00	0	0	0	0	74
3.00	2.00	0	0	0		52
1.00	2.00	0	0	0	0	62
1.00	1.00	0	0	0	0	40
1.00	1.00	0	0	0	0	55
1.00	1.00	0	0	0	0	41
2.00	1.00	0	0	0	0	28
2.00	3.00	0	0	0	0	45
1.00	1.00	0	0	0	0	26
3.00	2.00	0	0	0	0	78
1.00	1.00	0	0	0	0	60
2.00	0.00	0	0	0	0	31
1.00	3.00	0	0	0	0	66
3.00	4.00	0	0	0	0	76
2.00	0.00	0	0	0	0	44
1.00	1.00	0	0	0	0	53
1.00	2.00	0	0	0	0	27
2.00	1.00	0	0	0	0	81
2.00	3.00	0	0	0	0	59
1.00	0.00	0	0	0	0	86
1.00	1.00	0	0	0	0	51

1.00	1.00	0	0	0	0	37
2.00	0.00	0	0	0	0	20
3.00	2.00	0	0	0	0	75
3.00	1.00	0	0	0	0	61
1.00	1.00	0	0	0	0	60
2.00	1.00	0	0	0	0	68
1.00	1.00	0	0	0	0	45
1.00	0.00	0	0	0	0	50
3.00	2.00	0	0	0	0	76
2.00	3.00	0	0	0	0	74
1.00	3.00	0	0	0	0	60
2.00	1.00	0	0	0	0	61
1.00	3.00	0	0	0	0	75
2.00	1.00	0	0	0	0	36
2.00	2.00	0	0	0	0	63
3.00	1.00	0	0	0	0	66
2.00	1.00	0	0	0	0	47
3.00	3.00	0	0	0	0	87
1.00	4.00	0	0	0	0	60
1.00	3.00	0	1	0	0	89
2.00	0.00	0	1	0	0	49
1.00	1.00	0	1	0	0	48
1.00	1.00	0	1	0	0	49
3.00	1.00	0	1	1	0	82
1.00	3.00	0	1	0	0	67
1.00	3.00	0	1	0	0	33
3.00	1.00	0	1	0	0	68
1.00	2.00	0	1	1	0	54
1.00	1.00	0	1	1	0	57
1.00	4.00	0	1	0	0	52
3.00	2.00	0	1	1	0	66
2.00	4.00	0	1	0	0	58
2.00	0.00	0	1	1	1	70
2.00	2.00	0	1	0	0	28
1.00	1.00	0	0	0	0	73
2.00	4.00	0	0	0	0	85
1.00	1.00	0	0	0	0	50
1.00	3.00	0	0	0	0	59
2.00	4.00	0	0	0	0	27
1.00	1.00	0	0	0	0	85
2.00	3.00	0	1	0	0	55
2.00	1.00	0	1	1	0	59
2	1	0	1	0	1	61
1	3	0	1	0	0	75
1	0	0	1	0	0	68
1	1	0	1	0	0	56
2	1	0	0	0	0	84

2	0	0	0	0	0	49
1	0	0	0	0	0	44
1	1	0	0	0	0	52
1	1	0	0	0	0	75
1	0	0	0	0	0	41
2	4	0	0	0	0	78
2	1	0	1	0	0	45
1	3	0	0	0	0	60
3	1	0	0	0	0	74
1	0	0	0	0	0	67
1	4	0	0	0	0	54
2	0	0	0	0	0	44
1	0	0	0	0	0	51
1	2	0	0	0	0	42
1	1	0	0	0	0	51
1	1	0	0	0	0	57
1	2	0	0	0	0	69
1	1	0	0	0	0	28
1	3	0	0	0	0	54
1	1	0	0	0	0	85
1	0	0	0	0	0	55
1	2	0	0	0	0	53
1	1	0	0	0	0	25
2	3	0	0	0	0	76
1	1	0	0	0	0	36
1	1	0	0	0	0	68
1	1	0	0	0	0	53
1	4	0	0	0	0	81
1	4	0	0	0	0	60
1	3	0	0	0	0	65
1	3	0	0	0	0	72
1	0	0	0	0	0	71
1	2	0	0	0	0	77
1	2	0	0	0	0	62
1	4	0	0	0	0	85
1	1	0	DNR	0	0	87
1	2	0	1	1	0	42
1	4	0	DNR	0	0	61
1	1	0	DNR	0	0	73
1	2	0	0	0	0	71
1	0	0	0	0	0	42
1	2	0	0	0	0	67
1	4	0	DNR	1	0	91
1	4	0	1	0	1	82
2	2	0	0	0	0	44
2	0	0	0	0	0	90
3	1	0	0	0	0	27

2	0	0	0	0	0	89
2	2	0	0	0	0	31
1	1	0	0	0	0	25
1	1	0	0	0	0	61
1	1	0	0	0	0	47
1	1	0	0	0	0	58
2	4	0	0	0	0	64
2	3	0	0	0	0	76
1	3	0	0	0	0	68
1	2	0	0	0	0	70
1	4	0	0	0	0	73
3	0	0	0	0	0	48
1	1	0	0	0	0	44
1	2	0	0	0	0	56
1	3	0	0	0	0	92
1	2	0	0	0	0	28
1	3	0	0	0	0	41
1	1	0	0	0	0	46
2	1	0	0	0	0	65
2	0	0	0	0	0	58
1	3	0	0	0	0	81
1	0	0	0	0	0	59
1	1	0	0	0	0	47
1	3	0	0	0	0	49
1	0	0	0	0	0	40
1	1	0	0	0	0	54
2	1	0	0	0	0	64
2	1	0	0	0	0	46
2	4	0	0	0	0	67
3	0	0	0	0	0	56
1	0	0	0	0	0	45
1	3	0	0	0	0	42
2	1	0	0	0	0	52
1	0	0	0	0	0	66
1	1	0	0	0	0	70
1	3	0	0	0	1	69
1	1	0	0	0	0	79
1	2	0	0	0		57
2	3	0	1	0	0	71
2	4	0	1	0	1	65
3	0	0	0	0	0	75
1	4	0	DNR/DNI	0	0	95
2	1	0	1	1	0	55
2	3	0	0	0	0	62
1	1	0	0	0	0	26
3	2	0	0	0	0	53
1	0	0	1	0	0	43

2	2	0	0	0	0	73
3	2	0	DNR/DNI	0	0	69
2	1	0	0	0	0	70
2	3	0	DNR	0	0	98
2	2	0	0	0	0	67
1	2	0	0	0	0	76
1	1	0	0	0	0	42
1	3	0	0	0	1	60
2	1	0	1	1	0	64
2	1	0	1	0	0	78
2	4	0	DNR/DNI	0	0	94
1	0	0	1	0	0	65
1	2	0	0	0	0	64
2	3	0	1	1	0	62
1	2	0	1	1	0	64
2	3	0	1	0	0	46
1	3	0	0	0	0	36
1	0	0	1	1	0	60
2	2	0	0	0	0	58
1	3	0	1	0	0	48
1	1	0	0	0	0	55
1	1	0	1	0	0	35
1	4	0	1	1	0	67
2	0	0	1	1	0	72
3	0	0	DNR/DNI	0	0	79
1	0	0	1	0	0	53
1	3	0	1	0	0	58
1	1	0	1	0	0	78
1	3	0	1	0	0	44
1	2	0	0	0	0	24
1	2	0	1	0	0	59
1	1	0	0	0	0	65
1	3	0	1	0	0	74
1	2	0	1	0	1	56
1	2	0	1	0	0	85
1	2	0	1	0	0	66
1	1	0	1	0	0	64
2	2	0	0	0	0	77
1	3	0	1	0	0	53
2	1	0	0	0	0	65
1	2	0	1	0	0	70
2	2	0	1	0	0	39

Race (1=AA	Sex (0=mal	Smoking Ev	BMI	Obese (>/=	Ho Diabete	Ho HTN	Ho Asthma	Ho COPD
1	0	0	54	1	1	1	0	0
1	0	0	28.5	0	1	0	0	0
2	1		24.3	0	0	1	0	0
1	1	0	35.8	1	1	1	0	0
2	0	0	32.6	1	0	1	0	1
3	1	0	27.5	0	1	0	0	0
1	1	0	28.6	0	1	0	0	0
3	0	0	26.7	0	0	0	0	0
1	1	0	24.2	0	0	1	1	0
1	0	0	21.9	0	0	0	0	0
1	0	0	23.1	0	0	0	0	0
1	0	0	29.7	0	1	1	0	1
2	0	1	29.9	0	1	0	1	1
1	0		23.8	0	0	1	1	0
1	1	1	24.4	0	1	1	0	1
1	0		63.3	1	1	1	0	0
1	1	1	24.9	0	0	1	0	0
1	0	0	40.3	1	0	1	0	0
1	0	0	38.5	1	1	1	0	0
1	0	0	23.2	0	0	0	0	0
1	0	0	37.9	1	0	1	0	0
3	0	0	31.9	1	0	0	0	0
2	0	0	31.9	1	0	0	0	0
4	1	0	23.3	0	0	1	0	0
1	0	0	35.4	1	1	0	0	0
1	0	0	43	1	1	1	0	0
1	0	0	19.1	0	0	1	0	1
3	0	0	29.5	0	0	0	0	0
1	1		31.7	1	0	1	0	0
1	0	0	33.4	1	1	1	0	0
1	0	1	33.1	1	1	1	0	0
	1	0	44.5	1	0	0	0	0
3	0	0	39.6	1	0	0	1	0
3	1	0	24.1	0	1	1	0	0
1	0		26.4	0	1	1	0	0
1	1	1	36	1	0	0	0	0
1	0	0	23	0	0	0	0	0
1	1	0	38.1	1	0	0	0	0
1	1	0	96.9	1	0	1	1	0
3	0	0	62.4	1	0	1	0	0
	0	1	29.1	0	1	1	0	0
3	1	0	40.3	1	1	0	0	0
1	1	0	40.2	1	1	1	0	0
1	0	0	38	1	0	1	0	0
1	1	0	46.9	1	0	1	0	0
3	1	0	25.6	0	0	1	0	0

1	0	0	25.7	0	0	0	0	0
1	0	1	35.3	1	1	1	0	0
1	1	1	36.3	1	1	0	0	1
	1		19.8	0	0	0	0	0
1	1		37.5	1	0	0	0	0
1	0	0	27.1	0	0	0	0	0
2	1	1	21.7	0	0	0	0	0
1	1	0	43.7	1	0	1	0	0
1	0	1	27.2	0	0	1	0	1
1	0	0	34.3	1	0	0	0	0
2	1	0	51.9	1	0	1	0	0
	0	0	27.6	0	0	1	1	0
2	0	1	27.3	0	0	0	0	0
2	0	1	22.5	0	0	0	0	0
2	0	1	31	1	0	1	0	1
1	1	0	33.2	1	0	0	0	0
1	0		49.2	1	0	0	0	0
1	1	0		1	1	0	0	0
4	1	0	24.2	0	0	0	0	0
3	1	0	29.2	0	1	1	0	0
1	0	0	22.8	0	0	1	0	0
1	0	0	31.1	1	1	1	0	0
1	0	0	35.1	1	1	1	0	1
4	0	1	22.1	0	0	0	0	0
1	0	0	25.1	0	1	1	0	0
2	1	0	35.5	1	1	1	0	0
1	1	0	19.5	0	0	0	0	1
1	1	1	47.8	1	1	1	0	0
1	1	0	27.5	0	1	1	0	0
1	0	0	43.5	1	0	0	0	0
2	1	0	41.6	1	1	1	0	0
1	1		31.1	1	0	0	0	0
4	0	0	29	0	0	0	0	0
1	1	0	42.5	1	0	0	1	0
3	0	0	32.2	1	0	0	0	0
1	0	1	25.1	0	1	1	0	1
1	0	0	32.6	1	0	1	0	0
1	1	0	25.3	0	0	0	0	0
1	1	0	27	0	0	1	1	0
1	1	0	44.1	1	1	1	0	0
1	0	0	26.5	0	0	0	0	0
1	1	0	35.3	1	0	0	0	0
	0	1	29.7	0	0	0	0	0
2	0	0	19.2	0	1	0	0	0
1	1	0	31	1	1	1	0	0
2	0	0	32.1	1	1	1	0	0
1	0		29.7	0	0	1	0	0

1	1	0	46.7	1	0	1	1	0
1	1	0	38.1	1	0	0	0	0
	1	0	30.2	1	1	1	0	0
1	0	0	24.4	0	0	0	0	0
2	1	0	32.9	1	0	1	0	0
2	0	0	36.9	1	0	1	0	0
1	1	0	43.3	1	1	1	0	0
3	0	0	28.9	0	0	0	0	0
1	1	0	36.6	1	0	0	0	0
1	1	4	34.8	1	1	1	0	0
1	1	0	41.8	1	1	1	0	0
1	0	0	22	0	1	1	0	0
1	0	0	41.1	1	1	1	0	0
2	1		40.1	1	0	0	1	0
3	1	0	24.3	0	0	0	0	0
	0	0	22.9	0	0	0	0	0
	0	0	21.6	0	1	1	0	0
2	1	0	33.5	1	1	1	0	0
1	0		26.9	0	0	1	0	0
1	1	1	23.2	0	1	1	0	0
2	1	0	23.6	0	0	0	0	0
	0	0	25	0	0	0	0	0
1	0	0	35.7	1	0	1	0	0
3	0	1	28.3	0	1	1	0	0
1	1	0	30.1	1	1	1	0	0
1	1	0	34.5	1	0	0	0	0
1	1	1	27.3	0	0	1	0	0
1	1	0	52.7	1	0	1	0	0
1	1	0	54.3	1	0	1	0	0
1	0	0	59	1	0	0	0	0
3	0	1	33.5	1	0	1	0	0
2	0	0	46.1	1	1	1	0	0
1	0	1	27.8	0	1	1	0	0
1	0	0	66	1	0	0	1	0
1	1	0	41.3	1	0	1	0	0
1	1	0	26.2	0	0	1	0	0
1	1	0	44.3	1	1	1	0	0
1	1	0	39.1	1	1	1	1	0
1	0	0	23.7	0	0	1	0	0
2	1	0	25.1	0	1	1	1	1
1	1	1	33.7	1	0	1	1	0
1	1		37	1	1	1	0	0
2	0	0	21.6	0	0	0	0	0
1	1	0	27.5	1	0	1	0	1
1	1	0			1	1	0	0
1	0	0	26.9	0	0	1	0	0
2	1	1	31.3	1	1	1	0	0

1	1	0	47.4	1	1	1	0	0
1	0	0	46.2	1	1	1	0	0
3	0	0	27.1	0	0	0	0	0
1	1	0	35.9	1	1	1	0	0
	0	0	32.2	1	0	0	0	0
1	1	1	33.4	1	1	1	0	0
1	0	0	27.8	0	1	0	0	0
1	1	0	37	1	1	1	0	0
1	1	0	35	1	0	1	0	0
1	1	0	34.4	1	0	0	0	0
1	1	0	36.9	1	0	0	0	0
1	1	0	35.2	1	1	1	0	0
1	0	0	29.1	0	0	0	0	0
3	0	0	25.7	0	0	0	0	0
3	0	0	29.8	0	0	0	1	0
1	1	0	24.8	0	1	1	0	0
3	0	0	28.2	0	0	0	0	0
2	0	1	30.6	1	0	0	0	0
3	0	0	27	0	0	0	0	0
4	1	0	24.7	0	1	1	0	0
1	1	0	20.6	0	0	0	1	0
2	0	0	27.3	0	0	1	0	0
2	1	0	27.6	0	0	0	0	0
1	1	1	35.7	1	1	1	0	0
	1	0	35	1	0	0	1	0
1	0	0	30.5	1	0	0	0	0
1	0		29.4	0	0	1	1	0
1	1	0	22.7	0	0	1	0	0
3	1	0	34	1	0	0	0	0
	1	0	26.7	0	0	1	0	0
2	1	1	30.6	1	1	0	0	1
1	0	1	28.2	0	1	1	1	0
	1	0	25.9	0	1	1	0	0
1	0	1	37.4	1	0	1	0	0
2	1	1	19.6	0	1	1	0	0
1	1	1	20.9	0	1	1	0	0
2	0	0	36.6	1	0	1	0	0
3	1	1	35.6	1	1	1	0	0
2	1	0	27.5	0	0	0	0	0
3	0	1	34.5	1	0	1	0	1
1	1	0	39.2	1	0	1	0	0
1	0	0	30.2	1	1	1	0	0
2	1	0	17.8	0	1	1	0	0
1	1	0	25.8	0	1	1	0	0
1	0	0	40.2	1	0	0	0	0
2	1	0	33.3	1	0	1	0	0
1	0	1	20.5	0	0	0	0	0

2	0	0	25	0	0	1	0	0
3	0	1	28.5	0	0	0	0	0
3	0	0	28.7	0	0	1	0	0
3	0	0	41.7	1	1	1	0	0
1	1	0	38.7	1	0	0	0	0
1	0	0	25.3	0	1	1	0	0
1	0	1	24.1	0	1	1	0	0
2	0	1	28.4	0	1	1	0	0
1	0	1	23.6	0	0	0	0	0
4	0	0	29.2	0	1	0	0	0
1	0	1	20.1	0	0	1	0	1
1	0	0	32.9	1	1	1	0	0
3	0	1	30.5	1	0	1	0	0
3	0	0	28.4	0	0	0	0	0
1	1	0	28.4	0	0	1	0	1
1	1	1	27.5	0	0	0	0	0
3	0	0	40.7	1	0	0	0	0
1	1	0	36.5	1	0	0	0	0
1	1	1	42.1	1	0	1	0	1
1	0		32.2	1	0	0	0	0
1	1	0	30	1	1	1	0	0
1	1		43.6	1	0	0	0	0
2	0	0	37.8	1	1	1	0	0
2	1	1	38.3	1	1	1	1	0
3	0	0	23.7	0	1	0	0	0
3	0	0	21.1	0	1	0	0	0
3	1	0	31.3	1	0	0	0	0
3	1	0	35.2	1	1	0	0	0
1	1		29.3	0	0	0	0	0
3	1	0	28.2	0	0	0	0	0
1	0	0	12.1	0	0	1	0	0
2	1	0	34.5	0	0	0	0	0
1	1	0	39	1	0	0	0	0
3	1	0	27.2	0	1	1	0	0
1	1	0	40.4	1	1	1	0	0
1	1	1	30.1	1	1	0	0	0
1	0		23.2	0	0	0	0	0
	0	1	27.9	0	0	1	0	0
2	0	0	23.1	0	1	0	0	0
1	1	0	28	0	0	1	1	0
1	0	1	27.8	0	1	0	0	1
1	1				1	1	0	0
3	0	0	47.1	1	1	1	0	0
1	0	0	35.9	1	1	1	0	0
3	0	1	30.4	1	0	0	0	0
2	0	1	35.5	1	1	1	0	0
3	0		34.8	1	1	1	0	0

3	0		32	1	0	0	0	0
1	0	0	19.8	0	1	1	0	1
1	0	1	18.2	0	0	1	0	0
1	1		16.5	0	0	1	0	0
3	1	0	29.3	0	1	1	0	0
1	0	1	21.7	0	0	1	0	1
	1	0	41.3	1	0	0	0	0
1	0	0	25.2	0	0	1	0	0
1	0	0	31.6	1	1	1	0	0
2	0	0	21.1	0	1	1	0	0
1	1		39.9	1	0	1	0	0
4	0	0	24.8	0	0	0	0	0
1	0	1	25.3	0	1	0	0	0
	1	0	33.1	1	0	1	0	0
1	0	1	47	1	1	1	1	0
3	0	0	37.8	1	1	1	0	0
2	0	0	46.9	1	0	0	0	0
1	1	0	31.6	1	0	2	0	0
1	0	0	36.3	1	0	1	0	0
3	0	0	33	1	1	1	0	0
1	0	0	25.2	0	0	1	0	0
4	0	0	26.3	0	0	0	0	0
	1	1	30	1	1	1	0	0
1	0	0	28.2	0	1	1	0	0
2	1	1	29.6	0	0	0	0	0
2	0	0	33.8	1	0	0	0	0
1	1	0	28.6	0	1	1	1	0
4	0	1	23.5	0	1	1	0	0
1	0	0	47.1	1	0	0	0	0
3	1	0	24.2	0	1	0	1	0
2	0	0	25.9	0	0	0	0	0
1	1	0	39	1	0	0	0	0
1	1	1	18.9	0	0	1	0	1
3	1		39.4	1	0	1	0	0
2	1		24.5	0	0	1	1	0
	0	0	20.8	0	0	1	0	0
2	0	1	26.6	0	0	1	0	0
1	0	0	18.3	0	0	0	0	0
1	0	0	39	1	0	1	0	0
1	0	0	21.6	0	0	0	0	1
1	1	1	39.7	1	0	1	0	0
1	1	0	28	0	0	1	0	0

Ho HIV	ESRD on HI	Ho_VTE	Ho_Afib	Ho_CAD	Ho_CVA	Known acti	ICU (1=yes,	Length of I(
0	0	0	0	1	0	0	1	33
0	0	0	0	0	0	0	1	7
0	0	0	0	0	1	0	1	
0	0	0	0	0	0	1	1	
0	0	0	0	1	0	0	1	
0	0	0	0	0	0	0	1	
0	0	0	0	0	0	0	1	
0	0	0	0	0	0	0	1	
0	1	1	1	0	0	0	1	
0	0	0	0	1	0	0	0	
0	0	0	0	0	0	0	1	
0	0	0	0	1	1	0	1	13
0	0	0	0	0	0	0	1	45
0	0	0	0	0	0	0	1	
0	0	0	0	1	1	0	1	
0	1	0	0	0	0	0	1	
0	0	0	1	0	0	0	1	
0	0	0	0	0	0	0	1	6
0	0	0	0	0	0	0	1	31
0	0	0	0	0	0	0	1	18
0	0	0	0	0	0	0	1	37
0	0	0	0	0	0	0	1	
0	0	0	0	0	0	0	1	
0	0	0	0	0	0	0	0	0
0	0	0	0	0	0	0	1	23
0	0	0	0	0	0	0	1	
0	1	0	0	0	1	0	1	6
0	0	0	0	0	0	0	1	29
0	0	0	0	0	0	0	1	
0	0	0	0	0	0	0	1	
0	0	0	0	0	0	0	0	
0	0	0	0	0	0	0	0	0
0	0	0	0	0	0	0	1	
0	0	0	0	0	0	0	1	8
0	0	0	0	0	0	0	1	
0	0	0	0	0	0	0	1	
0	0	0	0	0	0	0	1	44
0	0	0	0	0	0	0	1	5
0	0	0	0	0	0	0	1	
0	0	0	0	0	0	0	1	
0	0	0	0	1	0	0	1	
0	0	0	0	0	0	0	1	27
0	0	0	0	1	0	0	1	
0	0	0	0	0	0	0	0	
0	0	0	0	0	0	0	1	
0	1	0	0	0	0	0	1	48

0	0	0	0	0	0	0	1	38
0	1	0	0	1	0	0	1	27
0	0	0	0	0	0	0	1	32
0	0	0	0	0	0	0	1	42
0	0	0	0	0	0	0	1	42
0	0	0	0	0	0	0	1	22
0	0	0	0	0	0	0	1	12
0	0	0	0	0	0	0	1	10
0	0	0	0	1	0	0	1	10
0	0	0	0	0	0	0	1	8
0	0	0	0	0	0	0	1	8
0	0	0	0	0	0	0	1	8
0	0	0	0	0	0	0	1	5
0	0	0	0	0	0	0	1	5
0	0	0	0	0	0	0	1	4
0	0	0	0	0	0	0	1	4
0	0	0	0	0	0	0	1	3
0	0	0	0	0	0	0	1	3
0	0	0	0	0	0	0	1	3
0	0	0	0	0	0	0	1	3
0	0	0	0	0	0	1	1	3
0	0	0	0	0	0	0	1	3
0	0	0	0	0	0	0	1	2
0	0	0	0	0	0	0	1	2
0	0	0	0	0	1	0	1	2
0	0	0	0	0	0	0	1	1
0	0	0	0	0	0	0	1	1
0	0	0	0	0	0	0	1	1
0	0	0	0	0	0	0	1	1
0	0	0	0	0	0	0	0	0
0	0	0	0	0	0	0	0	0
0	0	0	0	0	0	0	0	0
0	0	0	0	0	0	0	0	0
0	0	0	0	0	0	0	0	0
0	0	0	0	0	0	0	0	0
0	0	0	1	1	0	1	0	0
0	0	0	0	0	0	0	0	0
0	0	0	0	0	0	0	0	0
0	0	0	0	0	0	0	0	0
0	0	0	0	0	0	0	0	0
0	0	0	0	0	0	0	0	0
0	0	0	0	0	0	0	0	0
0	0	0	0	0	0	0	0	0
0	0	0	0	0	0	0	0	0
0	1	0	0	0	0	0	0	0
0	0	0	1	0	0	1	0	0
0	0	0	0	0	0	0	0	0

0	0	0	0	0	0	0	0	0
0	0	0	0	0	0	0	0	0
0	0	0	0	0	0	0	0	0
0	0	0	0	1	0	0	0	0
0	0	0	0	0	0	0	0	0
0	0	0	0	0	0	0	0	0
0	0	0	0	0	0	0	0	0
0	0	0	0	0	0	0	0	0
0	0	0	0	0	0	0	0	0
0	0	0	0	1	0	0	0	0
0	0	0	0	0	0	0	0	0
0	0	0	0	0	0	0	0	0
0	0	0	0	0	0	0	0	0
0	0	0	0	0	0	0	0	0
0	0	0	0	0	0	0	0	0
0	0	0	0	1	0	0	0	0
0	0	0	0	0	0	0	1	0
0	0	0	1	1	0	0	0	0
0	0	0	0	0	0	0	0	0
0	0	0	0	0	0	0	1	
0	0	0	0	0	0	0	1	6
0	0	0	0	0	0	0	1	7
0	0	0	0	0	0	0	1	9
0	0	0	0	1	1	0	1	9
0	0	0	0	0	1	0	1	6
0	0	0	0	0	0	0	1	11
0	0	0	0	0	0	0	1	15
0	0	0	0	0	0	0	1	14
0	0	0	0	0	0	0	1	15
0	0	0	0	0	0	0	1	21
0	0	0	0	0	0	0	1	20
0	0	0	0	0	0	0	1	19
0	0	0	0	0	0	1	1	36
0	0	0	0	0	0	0	1	54
0	0	0	0	0	0	0	1	8
0	0	0	0	0	0	0	1	1
0	0	0	0	0	0	0	0	0
0	0	0	0	0	0	0	0	0
0	0	0	0	0	0	0	0	0
0	0	0	0	0	0	0	0	0
0	0	0	0	1	0	0	1	5
0	0	0	0	1	1	0	1	16
0	0	0	0	0	0	0	1	
0	0	0	0	0	0	0	1	34
0	0	0	0	0	0	0	1	28
0	0	0	0	0	0	0	1	
0	0	0	1	0	1	0	0	

0	0	0	0	0	0	0	0
0	0	0	0	0	0	0	0
0	0	0	0	0	0	0	0
0	0	0	0	0	0	0	0
0	0	0	0	0	0	0	0
0	0	0	0	0	0	0	0
0	0	0	0	0	0	0	0
0	0	0	0	0	0	0	0
0	0	0	0	0	0	0	0
0	0	0	0	0	0	0	0
0	0	0	0	0	0	0	0
0	0	0	0	0	0	0	0
0	0	0	0	0	0	0	0
0	0	0	0	0	0	0	0
0	0	0	0	0	0	0	0
0	0	0	0	0	0	0	1
0	0	0	0	0	0	0	0
0	0	0	0	0	0	0	0
0	0	0	0	0	0	0	0
0	0	0	0	0	0	0	0
0	0	0	0	0	0	1	0
0	0	0	0	0	0	0	0
0	0	0	0	0	0	0	0
0	0	0	0	1	0	0	0
0	0	0	0	0	0	0	0
0	0	0	0	0	0	0	0
0	0	0	0	0	0	0	0
0	0	0	0	0	0	0	0
0	0	0	0	0	0	0	0
0	0	0	0	0	0	0	0
0	0	0	0	0	0	0	0
0	0	0	0	0	0	0	0
0	0	0	0	0	1	0	0
0	1	0	0	0	0	0	0
0	0	0	0	0	0	0	0
0	0	0	0	0	0	0	0
0	0	0	0	0	0	0	1
0	1	0	0	0	0	0	1
0	0	0	0	0	0	0	0
0	0	0	1	0	0	0	0
1	0	0	0	0	0	0	0
0	0	0	0	0	0	0	1
0	0	0	1	0	1	0	1
0	0	1	0	0	0	0	0
0	0	0	0	0	0	0	0
0	0	1	0	1	0	0	0
1	0	0	0	0	0	0	0

0	0	0	0	0	0	0	0
0	0	0	0	0	0	0	1
0	0	0	0	0	0	0	1
0	0	0	0	0	0	0	1
0	0	0	0	0	0	0	0
0	0	0	0	0	0	0	0
0	0	0	0	1	0	0	1
0	0	0	0	0	1	0	1
0	0	0	0	0	0	0	0
0	0	0	0	0	0	0	0
0	0	0	0	0	1	0	1
0	1	0	0	0	0	0	0
0	0	0	0	0	0	0	0
0	0	0	0	0	0	0	0
0	0	0	0	0	0	0	0
0	0	0	0	0	0	0	0
0	0	0	0	0	0	0	1
0	0	0	0	0	0	0	0
0	0	0	0	0	0	0	0
0	0	0	0	0	0	0	0
0	0	0	0	0	0	0	0
0	0	0	0	0	0	0	1
0	1	0	0	0	0	0	0
0	0	0	0	0	0	0	0
0	0	0	0	0	0	0	0
0	0	0	0	0	0	0	0
0	0	0	0	1	0	0	1
0	0	0	0	0	0	0	0
0	0	0	0	0	0	0	1
0	0	0	0	0	0	0	1
0	0	0	0	0	0	0	1
0	0	0	0	0	0	0	0
0	0	0	0	0	0	0	1
0	0	0	0	0	0	0	1
0	0	0	0	0	1	0	0
0	1	0	0	0	1	0	1
1	0	0	0	0	0	0	1
0	0	0	0	0	0	0	1
0	0	0	0	0	0	0	1
0	0	0	0	0	0	0	1
0	0	0	0	0	0	1	1
0	0	0	0	0	0	0	0
0	1	0	0	0	0	0	1
0	0	0	0	0	1	1	0
0	0	0	0	0	0	0	1
0	0	0	0	0	0	0	1
0	0	0	0	0	0	0	1

0	0	0	0	0	0	0	1
0	0	0	0	0	1	0	0
1	1	0	0	0	0	0	0
0	0	0	0	0	0	0	0
0	0	0	0	1	0	0	1
0	0	0	0	0	0	0	1
0	0	0	0	0	0	0	0
0	1	0	0	0	1	0	1
0	0	0	0	1	1	0	1
0	0	0	0	0	0	0	1
0	0	0	0	0	0	0	1
0	0	0	0	0	0	0	1
0	1	0	0	0	0	0	1
0	0	0	0	0	0	0	1
0	0	0	0	0	0	0	1
0	0	0	0	0	0	0	1
0	0	0	0	1	0	0	1
0	0	0	0	0	0	0	1
0	0	0	0	0	0	0	0
0	0	0	0	0	0	0	1
0	0	0	0	0	0	0	0
0	0	0	0	0	0	0	1
0	0	0	0	0	0	0	1
0	0	0	0	0	0	0	1
0	0	0	0	0	0	0	1
0	0	0	0	0	0	0	1
0	0	0	0	0	0	0	1
0	0	0	0	0	0	0	1
0	0	0	0	0	0	0	1
0	0	0	0	0	0	0	1
0	0	0	0	0	0	0	1
0	0	0	0	0	0	0	1
0	0	0	0	0	0	0	1
0	0	0	0	0	0	0	1
0	0	0	1	0	0	0	1
0	0	0	0	0	0	0	1
0	0	0	0	0	0	0	1
0	0	0	0	0	0	0	0
0	0	0	0	0	0	0	1
0	0	0	0	1	0	0	1
0	0	0	0	0	0	0	1
0	0	0	0	0	0	0	1

Intubation	Length of Intubation
1	
0	0
1	13
1	6
0	0
0	0
1	9
0	0
1	20
1	1
1	
1	6
0	0
0	0
1	
0	0
1	21
1	
1	

1	
1	
1	
1	
1	
1	0
0	0
0	0
0	0
0	0
0	0
0	0
0	0
0	0
0	0
0	0
0	0
0	0
0	0
0	0
0	0
0	0
0	0
0	0
	0
0	0
0	0
0	0
0	0
0	0
0	0
0	0
0	0
0	0
0	0
0	0
0	0
0	0
0	0
0	0
0	0
0	0
0	0
0	0
0	0
0	0
0	0

0	0
0	0
0	0
0	0
0	0
0	0
0	0
0	0
0	0
0	0
0	0
0	0
0	0
0	0
0	0
0	0
	0
0	0
0	0
1	
1	2
1	3
1	6
1	6
1	6
1	9
1	11
1	13
0	15
1	16
1	18
1	18
1	7
1	
0	0
0	0
0	0
0	0
0	0
0	0
1	5
1	8
	71
